# miR-1227 Targets SEC23A to Regulate the Shedding of Large Extracellular Vesicles

**DOI:** 10.3390/cancers13225850

**Published:** 2021-11-22

**Authors:** Andrew Chin, Javier Mariscal, Minhyung Kim, Giorgia Guerra, Blandine Victor, Chen Qian, Elisabetta Broseghini, Edwin Posadas, Michael R. Freeman, Shivani Sharma, Paolo Gandellini, Nadia Zaffaroni, Sungyong You, Keith Syson Chan, Jlenia Guarnerio, Muller Fabbri, Dolores Di Vizio

**Affiliations:** 1 Department of Surgery, Division of Cancer Biology and Therapeutics, Cedars-Sinai Medical Center, Los Angeles, CA 90048, USA; archelonian@gmail.com (A.C.); j.mariscal.avila@gmail.com (J.M.); Minhyung.Kim@cshs.org (M.K.); giorgia.guerra@cshs.org (G.G.); blandine.victor@cshs.org (B.V.); Chen.Qian@cshs.org (C.Q.); michael.freeman@cshs.org (M.R.F.); Sungyong.You@cshs.org (S.Y.); 2 Cancer Biology Program, University of Hawai’i Cancer Center, Honolulu, HI 96813, USA; EBroseghini@cc.hawaii.edu (E.B.); mfabbri@cc.hawaii.edu (M.F.); 3 Department of Biomedical Sciences, Division of Cancer Biology and Therapeutics, Cedars-Sinai Medical Center, Los Angeles, CA 90048, USA; Edwin.Posadas@cshs.org; 4 Samuel Oschin Comprehensive Cancer Institute, Division of Cancer Biology and Therapeutics, Cedars-Sinai Medical Center, Los Angeles, CA 90048, USA; KeithSyson.Chan@cshs.org (K.S.C.); jlenia.guarnerio@cshs.org (J.G.); 5 Department of Medicine, David Geffen School of Medicine, University of California Los Angeles, Los Angeles, CA 90095, USA; 6 Department of Pathology & Laboratory Medicine, California NanoSystems Institute, Jonsson Comprehensive Cancer Center, David Geffen School of Medicine at UCLA, University of California Los Angeles, Los Angeles, CA 90095, USA; sharmas@ucla.edu; 7 Department of Biosciences, University of Milan, 20122 Milan, Italy; paolo.gandellini@unimi.it; 8 Department of Experimental Oncology and Molecular Medicine, Fondazione IRCCS Istituto Nazionale Tumori, 20133 Milan, Italy; 9 Department of Applied Research and Technological Development, Fondazione IRCCS Istituto Nazionale Tumori, 20133 Milan, Italy; nadia.zaffaroni@istitutotumori.mi.it

**Keywords:** large oncosomes, extracellular vesicles, extracellular vesicle biogenesis

## Abstract

**Simple Summary:**

Extracellular vesicles (EVs) are heterogenous in size, cargo, and mechanism of biogenesis. While the mechanism of formation of small EVs, such as exosomes, has been widely investigated, little is known about the pathobiology of large EVs. We identify here a microRNA that alters cellular vesicologenesis increasing shedding of large EVs and slightly reducing shedding of small EVs. We also demonstrate that this microRNA, miR1227, targets SEC23A to promote this phenotype. Importantly, large EVs are released by cells undergoing a mesenchymal-amoeboid transition that functionally translates into a more metastatic phenotype.

**Abstract:**

Cancer cells shed a heterogenous mixture of extracellular vesicles (EVs), differing in both size and composition, which likely influence physiological processes in different manners. However, how cells differentially control the shedding of these EV populations is poorly understood. Here, we show that miR-1227, which is enriched in prostate cancer EVs, compared to the cell of origin, but not in EVs derived from prostate benign epithelial cells, induces the shedding of large EVs (such as large oncosomes), while inhibiting the shedding of small EVs (such as exosomes). RNA sequencing from cells stably expressing miR-1227, a modified RISCTRAP assay that stabilizes and purifies mRNA-miR-1227 complexes for RNA sequencing, and in silico target prediction tools were used to identify miR-1227 targets that may mediate this alteration in EV shedding. The COPII vesicle protein SEC23A emerged and was validated by qPCR, WBlot, and luciferase assays as a direct target of miR-1227. The inhibition of SEC23A was sufficient to induce the shedding of large EVs. These results identify a novel mechanism of EV shedding, by which the inhibition of SEC23A by miR-1227 induces a shift in EV shedding, favoring the shedding of large EV over small EV.

## 1. Introduction

Extracellular vesicles (EV) exert potent physiologic effects and mediate the intercellular transfer of biomolecules, such as protein and RNA. EV-mediated communication is altered in cancer cells to perturb pathways that facilitate cancer progression and metastasis. The transfer of cancer-associated micro-RNAs (miRNAs) in EVs has attracted great attention, due to the potential for alterations in protein translation in distant cells. We have previously found that miR-1227-3p (miR-1227) is one of the most enriched miRNAs in EVs, obtained specifically from transformed, tumorigenic prostate epithelial cells, in comparison to EV from an isogenic, non-tumorigenic prostate epithelial line [1]. miR-1227 has been shown to induce the migration of cancer-associated fibroblasts and glioblastoma cells [1,2]; however, its role in prostate cancer cells and its functional targets are completely unknown.

Most studies on the role of EVs in cancer have focused on small (~100 nm diameter) EVs, such as exosomes; however, cells shed a wide range of EV populations, differing in size and cargo, and the functional role of distinct EV populations in cancer is poorly understood [3]. EVs can be broadly categorized into 40–200 nm small EVs (S-EV), which include exosomes and small ectosomes, and significantly larger (1–10 µm) EVs (L-EV), including large oncosomes [3]. While S-EV are shed by virtually all cells in the body, large oncosomes are shed specifically by cells undergoing amoeboid migration, making their shedding largely specific to invasive cancer cells. In addition to the differences in the cells from which they are shed, S-EV and L-EV also differ in their biogenesis, and S-EV are generated through multiple mechanisms, including the inward budding of endosomal membranes to generate multi-vesicular bodies, followed by fusion of multi-vesicular bodies to the plasma membrane to release exosomes and direct budding from the plasma membrane to release small ectosomes. L-EV, such as microvesicles, large ectosomes, and large oncosomes, can be generated through direct budding of the plasma membrane through pathways that are still largely unexplored [4]. L- and S-EV originate from different molecular pathways and carry distinct cargo [5], suggesting that L- and S-EV should be differentially regulated in diseases, such as cancer. Here, we show that miR-1227 promotes L-EV shedding, which might be one of the mechanisms by which the miRNA promotes aggressive behavior. miR-1227 also inhibits, at least at a certain extent, the shedding of S-EV, suggesting that miR-1227 differentially regulates the shedding of L- and S-EV. We performed orthogonal studies to identify the direct targets of miR-1227 and demonstrated that miR-1227’s differential alteration on the shedding of L- and S-EV occurs by directly targeting the COPII vesicle protein SEC23A. SEC23A is known to be downregulated in prostate cancer, and its loss is a driver for metastasis. Our data suggests that this may be, in part, due to alterations in EV shedding.

## 2. Materials and Methods

### 2.1. Statistics

Unless otherwise specified, significance was calculated using a two-tailed student’s *t*-test. Each point in all graphs represents a biological replicate with the bar heights representing the mean of the biological replicates. Unless otherwise specified, error bars represent the standard deviation among the biological replicates. When possible, three biological replicates were performed; however, some experiments (as noted in the Figure Legends) only perform one biological replicate, due to the difficulty in obtaining the samples.

### 2.2. Cell Culture

PC3 (prostate cancer, human), RWPE-2 (prostate cancer, human), and U87 (glioma, human) cells were purchased from ATCC. PC3 cells were grown in DMEM, supplemented with 10% fetal bovine serum. RWPE-2 were grown in keratinocyte serum-free media. U87 cells were grown in MEM, supplemented with 10% fetal bovine serum. All cell lines were routinely monitored for mycoplasma contamination. PC3, RWPE-2, and U87 cells stably expressing miR-1227 were generated, as previously described [1]. PC3 cells stably expressing SEC23A shRNA were generated by lentiviral transduction with SEC23A MISSION shRNA (Sigma Aldrich, St. Louis, MO, USA; TRC# TRCN0000232509).

### 2.3. Patient Plasma

Blood samples were collected in BD Vacutainer™ ACD tubes and processed within 2 h of phlebotomy. Plasma was prepared from blood by centrifugation at 2000× *g*, 4 °C, for 5 min, followed by another spin at 2000× *g*, 4 °C, for 5 min. All human subject research was performed under the IRB study #PRO00033050, approved by the Cedars Sinai Medical Center ethics review board. Informed consent was obtained from all subjects. Pooled healthy human plasma was obtained from Innovative Research (Novi, MI, USA).

### 2.4. EV Isolation

S- and L-EV were isolated by differential ultracentrifugation, followed by density gradient purification, as previously described [6,7,8,9]. Cells were serum-starved for 24 h before isolation to avoid isolation of EV derived from FBS. For L-EV isolation from transiently transfected cells for tunable-resistance pulse sensing (TRPS) quantification, cells were plated into 6 well plates and serum-starved for 24 h. Conditioned media was collected and spun at 300× *g* for 5 min. The supernatant was transferred to a new tube and spun at 10,000× *g* for 30 min. The supernatant was discarded, and the pellet was resuspended in 80 µls PBS. The resuspended L-EV were spun again at 300× *g* for 5 min, and the supernatant was used for TRPS measurements.

### 2.5. EV Size and Concentration Measurements

TRPS was performed using a qNano Gold (Izon, Christchurch, New Zealand) with an NP250 nanopore (S-EV) or an NP2000 nanopore (L-EV), according to manufacturer instructions. Nanopores were stretched at 47 mm and voltage set either at 0.04 V for L-EV or 0.5 V for S-EV, in order to achieve a stable current baseline of about 120 nA. Particle size and concentrations were calibrated using Izon calibration particles (1:100 diluted TPK200 for S-EVs, and 1:1000 diluted CPC2000 for L-EVs) and a minimum of 500 events were registered for each sample, with a positive pressure of 5 mbar. For each biological replicate, at least 3 technical replicates were acquired, and the mean of the 3 highest technical replicates was used. Exclusion of data lower than the 3 highest technical replicates (for each biological replicate) was performed for all TRPS samples, due to the bias in potentially acquiring data from a nanopore that is partially clogged but does not show any deviation in quality control parameters (particle rate and amperage). This could result in undercounting the true particle concentration. False positives were identifiable, based on their unusual amperage deflection profiles, and samples with notable numbers of false positives were discarded pre-analysis. Thus, saved and analyzed data were biased to include more false negatives than false positives, so the highest 3 technical replicates were used to compensate for this. For atomic force microscopy (AFM) imaging and analysis, 5 µls of freshly isolated S- or L-EV samples were incubated on freshly cleaved mica substrates (TedPella Inc, Redding, CA, USA) for 5 min, washed with molecular grade de-ionized water, and imaged under ambient conditions. Samples were imaged by Dimension Icon (Bruker Instruments, Billerica, MA, USA) using TESP probes (Bruker Instruments, Billerica, MA, USA), and images were recorded in amplitude mode AFM at 512 samples per line at 1 Hz, as described previously [10,11]. Topographic height images were processed for zero order plane flattening using SPIPTM (Denmark). The particles were individually detected, and the diameter of the particles were quantified using the grain analysis function. All samples were stored at 4 °C and imaged within 24 to 48 h of isolation. 

### 2.6. Transwell Migration

RWPE-2 prostate cancer and U87 glioma cells stably expressing miR-1227 were seeded on to the upper well of 8 µm transwell inserts, followed by the addition of 10% FBS containing media to the lower chamber. After 6 h of incubation, at 37 °C, the transwells were fixed with 4% paraformaldehyde and stained with crystal violet. A cotton swab was used to remove cells on the top of the filter. The stained filters were washed three times in deionized water, and the filters were imaged on an all-in-one Keyence microscope. Image J was used to quantify the intensity of the stained cells that migrated to the bottom of the transwell. Intensity values were normalized to the corresponding vector control.

### 2.7. RNA Sequencing

RNA was isolated from RWPE-2 cells stably expressing miR-1227 or vector control using Trizol (Thermo Fisher, Waltham, MA, USA) for RNA sequencing. The RISCTRAP assay was performed as previously described [12]. For RNA-seq data analysis, the quality of sequence reads from the RNA-seq data were assessed, and low-quality reads were filtered using the FastQC tool (Babraham Bioinformatics, Cambridge, UK). Sequence alignment and quantification were performed using the STAR-RSEM pipeline [13,14]. Genes were filtered out and excluded from downstream analysis if they failed to achieve raw read counts of at least 2 across all the libraries. The RNA-seq data from stable cells were normalized by the quantile normalization method to reduce systemic bias between samples. The RNA-seq data from RISCTRAP samples were generated from the subset of mRNA, which was trapped by RISCTRAP method. It cannot be assumed that the complexity and total amount of the mRNA trapped by the control miRNA and the mRNA trapped by one miRNA are the same, which is a basic assumption of normalization. Therefore, we estimated the ratio of RISCTRAP miR-1227 and RISCTRAP control miRNA, as proposed by Beissbarth et al. and William et al. [15,16]:

$$R_{RC}=log_{2}\left[\left(n_{2}+f\right)/\left(n_{1}+f\right)\right]+log_{2}\left[\left(t_{1}-n_{1}+f\right)/\left(t_{2}-n_{2}+f\right)\right]\;$$

where, for each gene, R_RC_ (ratio of read count) is the log_2_ ratio of abundance between samples 1 and 2; n_1_ and n_2_ are read counts for the genes in sample1 and 2, respectively; t_1_ and t_2_ are total numbers of read count over all genes in the sample1 and 2. Enrichment analysis of gene ontology biological processes (GOBPs) was performed using the DAVID software [17]. The RNA seq data has been uploaded to the National Center for Biotechnology Information Sequence Read Archive (NCBI-SRA), BioProject accession number: PRJNA693580.

### 2.8. Quantitative PCR

RNA was isolated using Trizol (Thermo Fisher), and quantitative PCR was performed as previously described [1]. The concentration of miR-1227 in patient plasma was calculated using a standard curve of mirVana miR-1227 mimic (Thermo Fisher), ranging from 1 aM to 100 µM.

### 2.9. Identification of Targets of miR-1227 in Prostate Cancer

To define the target of miR-1227 function in prostate cancer, we used three independent data: (1) stable cell RNA-seq data, (2) RISCTRAP RNA-seq data, and (3) miRNA target prediction database. The genes downregulated more than 2-fold in miR-1227 overexpressing stable cells, compared to control vector treated cells that were defined as the potential targets of miR-1227. Next, the potential miR-1227 target genes in prostate cancer from RISCTRAP were defined as the following steps: (1) we estimated R_RC_ of RISCTRAP miR-1227, compared to control in normal and cancer cell line, and (2) genes with an R_RC_ value greater than 1.67, the top 5% of the R_RC_ estimated from the normal cell, were selected as potential miR-1227 target genes. Lastly, we collected the predicted miR-1227 target genes in at least 4 out of 5 independent miRNA target prediction algorithms (miRanda [18], miRDB [19], miRWalk [20], PICTAR5 [21], and Targetscan [22]) and defined the potential target genes of miR-1227 by selecting genes predicted by at least 4 of the 5. Finally, we defined common genes of these three analyses as target genes of miR-1227 in the prostate cancer.

### 2.10. Immunoblotting

Immunoblotting was performed as previously described [9]. Primary antibodies against SEC23A (8162) and HSPA5 (3177) were obtained from Cell Signaling Technology (Danvers, MA, USA). The antibody against β-actin (A5441) was obtained from Sigma Aldrich (St. Louis, MO, USA). Antibodies against KRT18 (ab93741) and CD81 (ab79559) were obtained from Abcam (Cambridge, United Kingdom). The antibody against CD9 (sc-13118) was obtained from Santa Cruz Biotechnology. The original blots data is shown in Figure S2.

### 2.11. Generation of SEC23A shRNA Stable Cell Lines

The lentiviral Mission pLKO.1-puro Vector SEC23A shRNA (TRCN0000232509) and control (SHC202) constructs were purchased from Sigma Aldrich. Stable cells lines were generated by co-transfecting HEK293T cells with the shRNA, packing (psPAX2), and envelope (pMD2.G) plasmids. After 72 h, the conditioned media containing lentivirus was strained through a 0.45 µm filter and stored at −80 °C for further use. To generate stable cell lines, PC3 or RWPE-2 cells were cultured in 6 cm dishes, until ~50% confluent, followed by transduction with shRNA lentivirus, supplemented with 10 µg/mL polybrene. After 72 h, the media was replaced with DMEM 10% FBS supplemented with 1 µg/mL puromycin.

### 2.12. SEC23A 3′UTR Luciferase Reporter Constructs

To the generate the SEC23A 3′UTR luciferase reporter construct, the SEC23A 3′UTR was amplified from RWPE-1 DNA using an Xho1 forward primer (CTCGAGGCTAATAATGTTAAAGACACT) and EcoR1 reverse primer (GAATTCGCCTAGCGTAGTTCAATTC) and cloned into the psiCHECK-1 luciferase reporter vector (Promega) using the inserted Xho1 and EcoR1 sites. The Q5 Site-Directed Mutagenesis kit (NEB) was used to delete the 2 predicted miR-1227 binding sites in the SEC23A 3′UTR (Figure S1C). Site A deletion primers: forward: GCAGACAGACCCAAATGAATTTTCTTGCTCACC, reverse: GGTGAGCAAGAAAATTCATTTGGGTCTGTCTGC. Site B deletion primers: forward: AAAATAAATGGTTTTGTTGGAG, reverse: TTTAAAAAAACTACTGCGTTTAG.

## 3. Results

### 3.1. MiR-1227 Induces Migration and Alters EV Shedding in Cancer Cells

**Figure 1:**
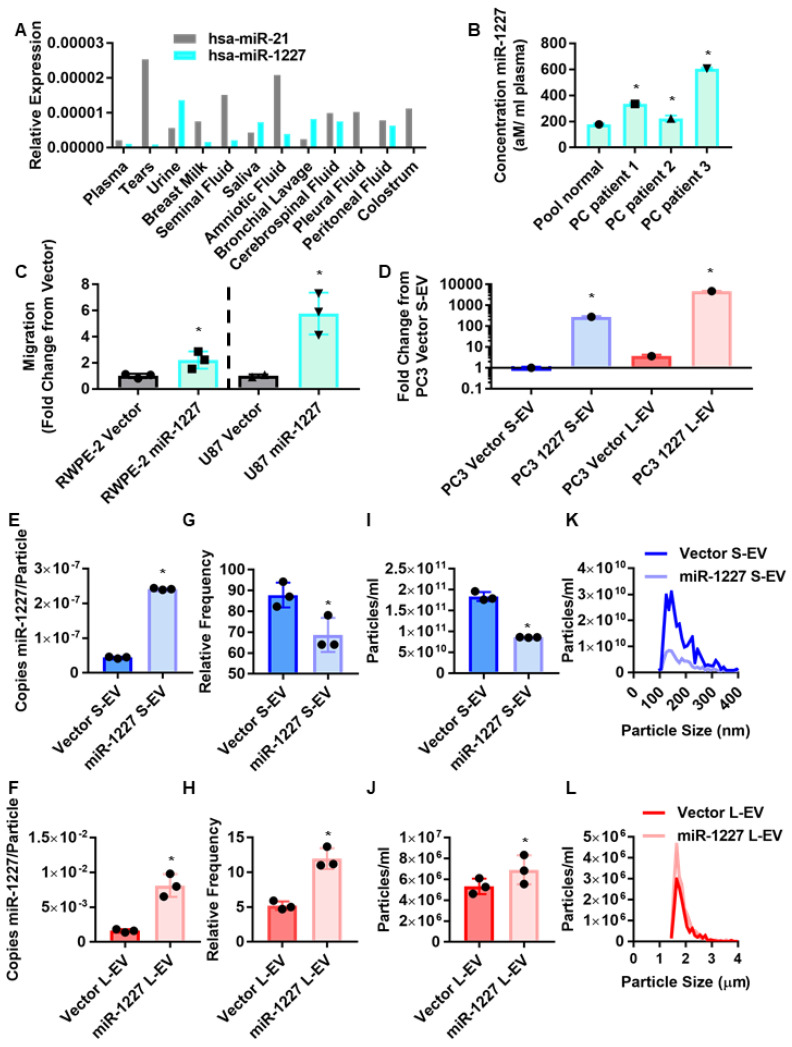
MiR-1227 induces shedding of L-EV and inhibits the shedding of S-EV in PC cells. (**A**) MiR-1227 (teal) and miR-21 (grey) expression in different biological fluids from Weber et al. 2010 [23]. (**B**) Taqman qPCR quantification of miR-1227 in EV isolated from pooled normal or PC patient plasma (*n* = 1). (**C**) Transwell migration of RWPE-2 PC and U87 glioma cells stably expressing miR-1227 (teal) or empty vector control (grey) (*n* = 3) normalized to corresponding vector control; (**D**) qPCR for miR-1227 from S-EV (blue) and L-EV (red) isolated from PC3 cells stably expressing miR-1227(*n* = 1). (**E**,**F**) Digital droplet qPCR quantification of miR-1227 per S-EV (**E**) and L-EV (**F**) (*n* = 3). (**G**,**H**) Atomic force microscopy quantifications of S-EV (**G**) and L-EV (**H**) shed from PC3 cells stably expressing miR-1227 (*n* = 3). (**I**–**L**) Quantification of S-EV (**I**) and L-EV (**J**) shed from PC3 cells stably expressing miR-1227 by TRPS with size distribution plots in (**K**,**L**), respectively (*n* = 3); * *p* < 0.05.

MiR-1227 was previously identified as one of the most enriched miRNAs in prostate cancer EV, compared to the originating prostate cancer cells but not in EV from isogenic benign prostate epithelial cells [1]. Although miR-1227 is poorly studied, and not frequently identified in prostate cancer databases, analysis of a publicly available dataset of miRNAs in human biofluids [23] indicated that miR-1227 is found at comparable abundance as another EV miRNA miR-21, which has been widely studied and consistently identified in the plasma of several biofluids [24,25,26,27,28,29] (supervision A). Interestingly, miR-1227 is notably more abundant in urine than miR-21, suggesting that miR-1227 may be particularly important for prostate cancer. This prompted us to investigate whether miR-1227 can be found in EVs isolated from the plasma of prostate cancer patients. Plasma EVs isolated from prostate cancer patients by differential centrifugation contained significantly more miR-1227 than plasma EVs isolated from pooled healthy donors (Figure 1B), indicating an association between the expression of miR-1227 and prostate cancer. Additionally, the stable expression of miR-1227 increased the migration of the poorly migratory cancer cell lines RWPE-2 and U87 (Figure 1C), suggesting a role for miR-1227 in tumor progression.

Most studies on the role of miRNAs in EVs have focused on miRNAs carried by S-EV, such as exosomes. However, the role of miRNAs in L-EV is poorly understood. Due to their larger size, L-EV could potentially carry substantially more miRNAs than S-EV. Therefore, we assessed the abundance of miR-1227 in L- and S-EV isolated from prostate cancer cells stably expressing miR-1227. We used differential ultracentrifugation, followed by density gradient purification, the gold standard methodology for isolating L- and S-EV [6,30]. The stable expression of miR-1227 in PC3 cells increased miR-1227 levels in both L- and S-EV (Figure 1D). Interestingly, we found that miR-1227 is ~16 fold more abundant in L-EV than S-EV, in line with the significantly larger volume and carrying capacity of L-EV and our previous findings showing that L-EV contain more RNA cargo than S-EV [5]. This was further confirmed by the digital droplet PCR quantification of the number of copies of miR-1227 in L- and S-EV, indicating that there are ~30,000 more copies of miR-1227 per L-EV than per S-EV (Figure 1E,F).

We characterized EV markers in EV isolated from miR-1227 overexpressing cells and surprisingly found that L-EV proteins, such as HSPA5 and KRT18, were increased in L-EV isolated from miR-1227 overexpressing cells, in comparison to control cells (Figure S1A), suggesting an increased number of L-EVs in miR-1227 overexpressing cells. This prompted us to investigate whether miR-1227 can alter the shedding L-EV. One of the challenges of comparing S- and L-EV is that many commonly used methods, such as nanoparticle tracking analysis and transmission electron microscopy, do not have a resolution window large enough to visualize both L- and S-EV. Therefore, we used atomic force microscopy (AFM), which can alter its resolution window through the use of different size probes, allowing for the quantification of both L- and S-EV. Surprisingly, AFM showed an increase in the number of L-EV, and a reduction in the number of S-EV derived from PC3 cells stably expressing miR-1227 (Figure 1G,H and Figure S1B). These results suggest that miR-1227 alters vesicle biogenesis pathways to promote the shedding of L-EV, while inhibiting the shedding of S-EV.

To further demonstrate the shift in EV shedding, observed by AFM, we used tunable-resistance pulse sensing (TRPS), which allows for the detection of a wide range of different particle sizes, through the use of different size nanopores. TRPS findings were consistent with results from AFM, confirming that miR-1227 increases the shedding of L-EV, while decreasing the shedding of S-EV (Figure 1I,J). Despite this shift in the total number of L- and S-EV, the size distribution of the EV was not altered (Figure 1K,L). To our knowledge, this is the first report of a miRNA differentially altering the shedding of two distinct EV populations. L-EV are shed from highly invasive cancer cells undergoing amoeboid migration [31,32,33,34]; consequently, this shift in EV shedding patterns may indicate an increase in the metastatic potential of cancer cells. This is in line with the increased migration of the poorly migratory RWPE-2 prostate cancer cells and U87 glioma cells, by the stable expression of miR-1227 (Figure 1C).

### 3.2. SEC23A Is a Direct Target of miR-1227

**Figure 2:**
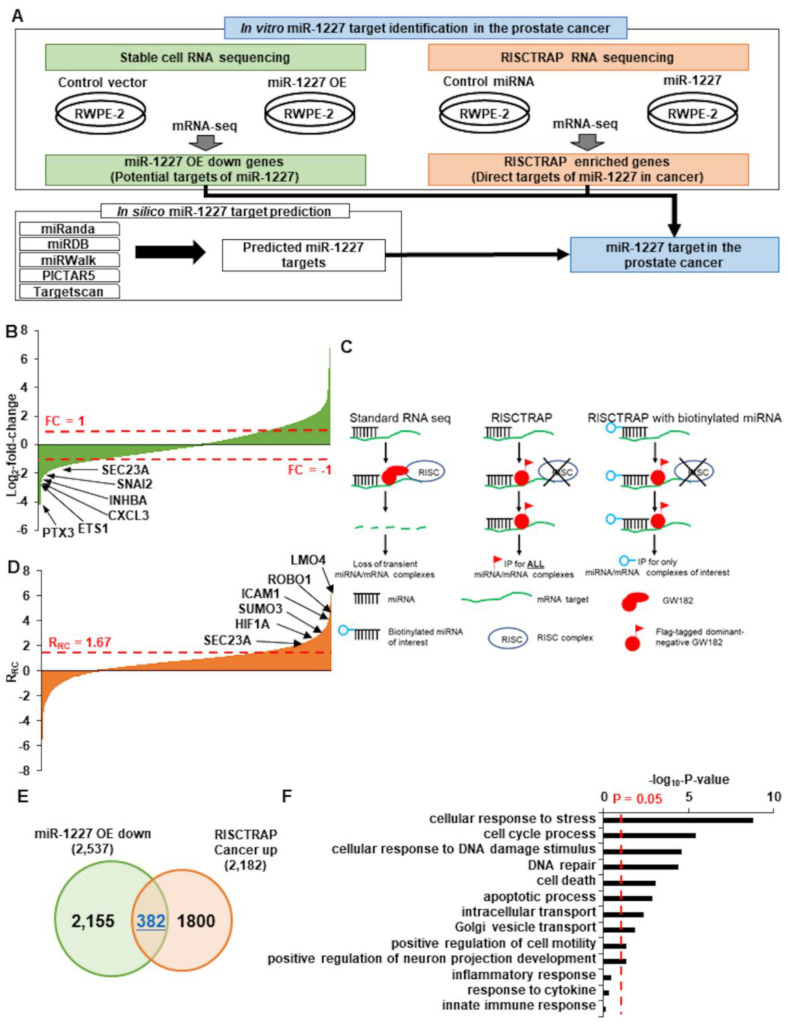
Identification of miR-1227 targets. (**A**) Summary of the bioinformatics strategy used to identify miR-1227 targets. (**B**) Waterfall plot of putative miR-1227 targets, identified by stable cell RNA sequencing in RWPE-2 cells stably expressing miR-1227. Prostate cancer-related genes downregulated by miR-1227 are highlighted. (*n* = 1) (**C**) Schematic of the modified RISCTRAP assay. In standard RNA sequencing, some mRNA targets are rapidly degraded by the miRNA and are, thus, not detectable by RNA sequencing. The RISCTRAP assay stabilizes mRNA-miRNA complexes with a FLAG-tagged dominant negative GW182 protein, trapping the mRNA-miRNA complexes in an inactive state. The cells are transfected with a miRNA of interest, and all mRNA-miRNA complexes are pulled down using FLAG for RNA sequencing. We modified this assay by transfecting the cells with a biotinylated miR-1227, which is used to specifically isolate the mRNA-miR-1227 complexes. (**D**) Waterfall plot of putative miR-1227 targets, identified by the modified RISCTRAP RNA sequencing. Select high confidence genes with an R_RC_ over 1.67 that are altered in prostate cancer are highlighted. (*n* = 1) (**E**) Overlap of the genes identified in the stable cell and RISCTRAP RNA sequencing experiments. Shared genes identified in both miR-1227 stable cell and RISCTRAP sequencing from cancer cells are in blue. (**F**) Gene ontology of the shared miR-1227 target genes.

In order to investigate how the poorly studied miRNA, miR-1227, may differentially alter the shedding of EVs, three independent, orthogonal methods were used to identify putative miR-1227 targets (Figure 2A). First, we performed RNA sequencing from RWPE-2 prostate cancer cells stably expressing miR-1227. Since most miRNA-targeted mRNAs are degraded following translation inhibition, we focused on those mRNAs that were at least two-fold down-regulated, in response to miR-1227 expression. This resulted in the identification of genes, such as PTX3, ETS1, and SEC23A, as downstream targets of miR-1227 (Figure 2B). Since this approach is unable to differentiate between direct and indirect functions of miR-1227, we employed a modified RISCTRAP assay [12,35] to identify the direct binding partners of miR-1227. In short, cells were transfected with a dominant negative GW182 protein to stabilize mRNA-miRNA complexes and a biotinylated miR-1227, which was used to specifically pulldown mRNA-miR-1227 complexes for RNA sequencing (Figure 2C). Several cancer-associated mRNAs were found using the RISCTRAP assay, including ICAM1, SUMO3, HIF1A, and SEC23A (Figure 2D; see details in Supplementary methods). We focused on the 382 mRNAs that were identified by both RNA sequencing experiments (Figure 2E). Gene ontology analysis from these shared genes identified pathways related to cell cycle, DNA damage, and Golgi vesicle transport (Figure 2F). High-confidence, prostate cancer-related miR-1227 targets were identified from these overlapping mRNAs by selecting for transcripts predicted to be miR-1227 targets by at least four of the following target prediction algorithms: miRanda [18], miRDB [19], miRWalk [20], PICTAR5 [21], and Targetscan [22]. We prioritized those targets previously linked to prostate cancer (Table S1).

**Figure 3:**
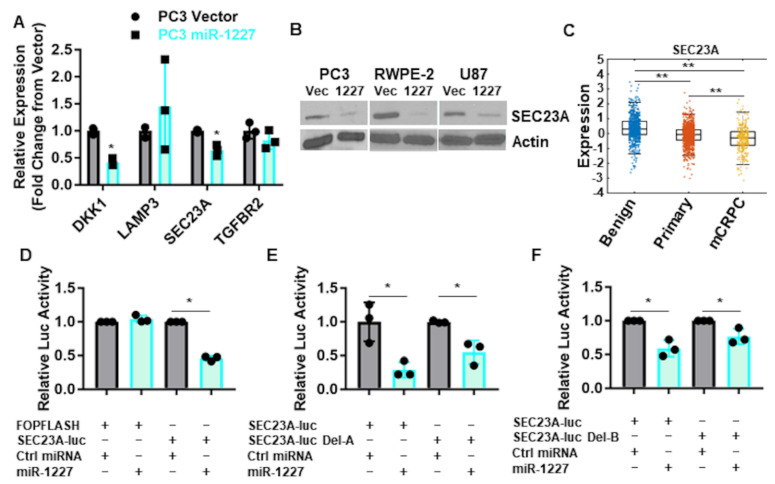
SEC23A is a direct target of miR-1227; (**A**) qPCR for high confidence miR-1227 target genes involved in vesicle formation from PC3 cells stably expressing miR-1227 (*n* = 3). (**B**) Western blot validation of SEC23A reduced expression in PC3, RWPE-2, and U87 cells stably expressing miR-1227. (**C**) Prostate cancer transcriptome atlas analysis of SEC23A in prostate cancer, showing reduced expression levels of SEC23A in cancer versus benign prostate tissue and in metastatic castration resistant versus primary prostate cancer. (**D**–**F**) Luciferase assays from RWPE-2 cells transfected with miR-1227 or control miRNA, plus the SEC23A 3′UTR luciferase construct (**D**) or the SEC23A 3′UTR luciferase construct with the deletion of 2 different predicted miR-1227 binding sites (**E**,**F**) (*n* = 3); * *p* < 0.05; ** *p* < 0.005.

To identify miR-1227 targets that could influence the shedding of EV, we queried the literature for these high-confidence miR-1227 targets to identify genes associated with vesicle trafficking, narrowing this list to four genes: DKK1, SEC23A, LAMP3 (CD63), and TGFBR2. Out of these genes, only DKK1 and SEC23A were validated to be decreased by qPCR from transiently transfected RWPE-2 cells (Figure 3A). We focused on SEC23A because it was consistently reduced at the protein level, in response to stable expression of miR-1227 in RWPE-2 and PC3 prostate cancer, as well as in U87 glioma cell lines (Figure 3B). Importantly, the inhibition of SEC23A has been shown to promote prostate cancer metastasis [36,37,38], and SEC23A expression is decreased in tumor tissues of patients with metastatic castration-resistant disease, in comparison to primary prostate cancer and benign tissue [39] (Figure 3C). Taken together, our RISCTRAP assay and in silico target analysis suggests that SEC23A is a direct target of miR-1227. To validate whether miR-1227 directly binds to the SEC23A 3′UTR, we cloned this region into a luciferase reporter construct. MiR-1227, but not a negative control miRNA, reduced the luciferase activity of the SEC23A 3′UTR reporter vector but not an unrelated luciferase construct (FOPFLASH) (Figure 3D). We identified two predicted binding sites for miR-1227 within the SEC23A 3′UTR (binding sites A and B, Figure S1C). Deletion of either of these binding sites partially abolished the regulation of the SEC23A 3′UTR luciferase construct by miR-1227 (Figure 3E,F). Taken together, these results suggest that miR-1227 binds directly to the 3′UTR of SEC23A to inhibit its translation.

### 3.3. Inhibition of SEC23A Induces the Shedding of L-EV

**Figure 4:**
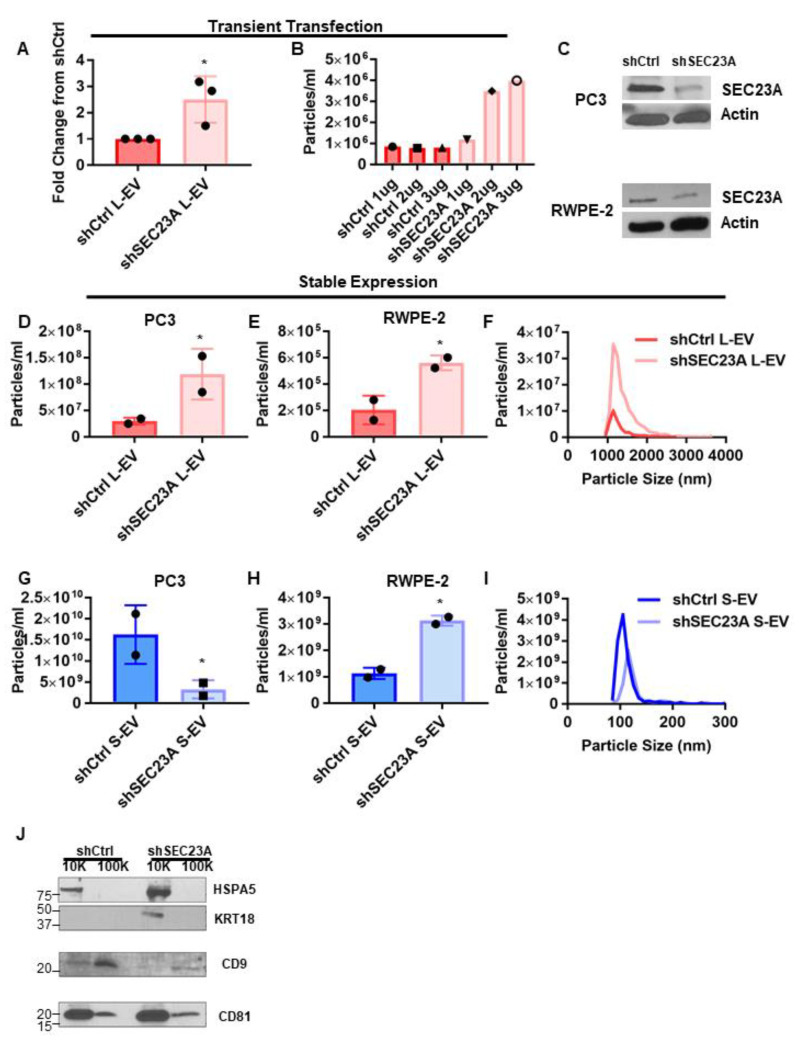
Inhibition of SEC23A induces L-EV shedding. (**A**) Quantification of L-EV shedding by TRPS in PC3 cells transiently transfected with SEC23A shRNA (*n* = 3). (**B**) Quantification of L-EV shedding by TRPS after transient transfection with increasing doses of SEC23A shRNA (*n* = 1). (**C**) Expression of SEC23A in PC3 cells stably expressing SEC23A shRNA. (**D**–**I**) Quantification of L-EV (**D**,**E**) and S-EV (**G**,**H**) shedding by TRPS in PC3 (**D**,**G**) and RWPE-2 (**E**,**H**) cells stably expressing SEC23A shRNA (*n* = 2). (**H**,**I**) Size distribution of L-EV (**F**) and S-EV (**I**) from PC3 cells stably expressing SEC23A shRNA. (**J**) Western blot for EV markers from EV isolated from PC3 cells stably expressing SEC23A shRNA; * *p* < 0.05.

To assess whether the inhibition of SEC23A is sufficient to alter EV shedding, we transiently silenced SEC23A in PC3 cells with SEC23A shRNA. Importantly, knockdown of SEC23A induced the shedding of L-EV (Figure 4A), thus phenocopying the result obtained with miR-1227 overexpression, suggesting that miR-1227 influences EV shedding by inhibiting expression of SEC23A. Transfection of increasing amounts of SEC23A shRNA induced an increase in L-EV shedding that was proportional to the knockdown of SEC23A by shRNA (Figure 4B), providing further evidence that increased L-EV shedding is a direct consequence of the inhibition of SEC23A. As a complementary approach, we knocked down SEC23A, by stably expressing SEC23A shRNA in PC3 and RWPE-2 cells (Figure 4C). Stable knock down of SEC23A also induced the shedding of L-EV in both PC3 and RWPE-2 cells (Figure 4D,E). Similar to the observations with miR-1227 overexpression, despite the alteration in the number of EV shed, inhibition of SEC23A did not alter the size profile of L-EV, suggesting that inhibition of SEC23A only alters the number of EV shed but not their physical properties (Figure 4F). Stable expression of SEC23A shRNA inhibited the shedding of S-EV in PC3 cells but, surprisingly, induced the shedding of S-EV in RWPE-2 cells (Figure 4G,H), suggesting that the regulation of S-EV shedding by SEC23A is context-dependent. Similar to the findings with L-EV, the inhibition of SEC23A did not notably alter the size distributions of S-EV, only the number of S-EV shed (Figure 4I), nor did it alter the expression of L- and S-EV protein markers (Figure 4J). Surprisingly, CD81 was more enriched in L-EV than S-EV, despite its frequent use as a S-EV marker. However, further experiments would need to be performed to determine whether this observation is also true in other cell lines. Together, this suggests that miR-1227 is a novel regulator of EV shedding that induces the shedding of L-EV through the inhibition of SEC23A.

## 4. Discussion

A few reports suggest a functional role for miR-1227 in cancer (in glioblastoma, in particular) [2]. In prostate cancer, miR-1227 has been identified as a non-coding RNA that is enriched in EVs from tumorigenic cells but not in EVs from an isogenic, non-tumorigenic cell line. Here, we show that miR-1227 is present at discrete levels in biological fluids and is increased in patients with aggressive prostate cancer, in comparison to patients with indolent or benign disease, suggesting a role in prostate cancer progression.

One of the seminal findings of this study is that miR-1227 is capable of differentially altering the shedding of distinct EV populations, favoring the shedding of L-EV. As the L-EV of the large oncosomes type are shed by highly invasive, amoeboid-migrating cells, the induction of L-EV shedding by miR-1227 suggests that this miRNA may induce the development of a highly invasive phenotype in cancer cells [31]. In contrast to L-EV, virtually all cells in the body shed S-EV. The functional role of these S-EV, in most healthy tissues, is poorly understood; however, there is increasing evidence that S-EV are important for protecting various organs from injury [40,41,42,43]. Furthermore, S-EV from stromal cells have been shown to induce dormancy in cancer cells [44,45]. While dormancy has been viewed as a means of evading chemotherapy, it could also be a mechanism used by the microenvironment to suppress metastasis [46]. S-EV shed by the cells in the microenvironment may induce dormancy in disseminated cancer cells, keeping them in check as occult tumors and preventing their growth into metastases. Cancer cells may transfer miR-1227 to stromal cells to hinder their ability to suppress cancer growth by inhibiting S-EV shedding, thus allowing these occult tumors to develop into metastases. However, further studies are required to see if this is the case.

We found here that miR-1227 is more highly expressed in L-EV than S-EV. This is an interesting finding, in consideration of the widely accepted knowledge that miRNAs are primarily shed in S-EV derived from the endosomal machinery, which suggests that mechanisms still largely unknown might modulate the export of select cargo in EVs.

We demonstrate that the induction of L-EV shedding by miR-1227 is mediated by the inhibition of the COPII vesicle protein SEC23A. Interestingly, the inhibition of SEC23A has been shown to promote prostate cancer metastasis through alterations in the secretome [36,37,38,47,48]. Here, we show that the inhibition of SEC23A phenocopies miR-1227 overexpression and induces the shedding of L-EV, suggesting this as an additional novel mechanism for promoting metastasis. Furthermore, the inhibition of SEC23A is known to induce cell migration. Combined with the increase in L-EV shedding, this may indicate that the inhibition of SEC23A induces an amoeboid phenotype, which is characterized by high invasiveness and increased shedding of L-EV. Further studies will investigate whether the resulting increased L-EV shedding is sufficient to promote metastasis. Interestingly, we observed that the inhibition of SEC23A has a context-dependent effect on the shedding of S-EV. This suggests that miR-1227 likely regulates S-EV shedding through additional targets aside from SEC23A. Additional investigation into how miR-1227 regulates S-EV shedding could provide insights into S-EV biogenesis.

## 5. Conclusions

We show that miR-1227, which is enriched in L-EV from prostate cancer cells and induces migration of poorly migratory cancer cells, targets SEC23A to induce the shedding of L-EV, while possibly inhibiting the shedding of S-EV. To our knowledge, this is the first report of a differential regulation of L-EV versus S-EV, which might have important functional consequences on tumor progression. miR-1227 and inhibition of SEC23A may prove to be valuable research tools to dissecting the specific roles of L- and S-EV in cancer metastasis.

## Data Availability

RNA sequencing data are available at BioProject Database, BioProject ID: PRJNA693580, http://www.ncbi.nlm.nih.gov/bioproject/693580.

## Supplementary Materials

The following are available online at https://www.mdpi.com/article/10.3390/cancers13225850/s1, Figure S1: miR-1227 increases L-EV markers and predicted binding sites in the SEC23A 3′UTR. (A) Western blot for EV markers from EV isolated from PC3 cells stably expressing miR-1227 or vector control. (B) AFM images, corresponding to the quantification in Figure 1G,H and showing EV isolated from PC3 cells stably expressing miR-1227. Scale bars represent 200 nm (S-EV) and 1 µm (L-EV). (C) The 2 predicted miR-1227 binding sites in the SEC23A 3′UTR that were deleted in luciferase assays. Binding site “A” is highlighted in green, while binding site “B” is highlighted in yellow. Figure S2: The original blots data. Table S1: high confidence miR-1227 targets in prostate cancer. High confidence miR-1227 targets were identified as being downregulated in RNA sequencing from cell stably expressing miR-1227, enriched in the modified RISCTRAP and identified by at least 4/5 in silico target prediction algorithms.
