# Supplementary material for: miR-1227 Targets SEC23A to Regulate the Shedding of Large Extracellular Vesicles
Source: Cancers (Basel). 2021 Nov 22;13(22):5850. doi: 10.3390/cancers13225850 (PMC8616086; doi:10.3390/cancers13225850)
Supplement: Supplementary file 1 [file cancers-13-05850-s001.zip › Supplementary Figure S1.pdf]

A

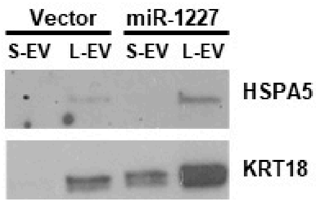

B

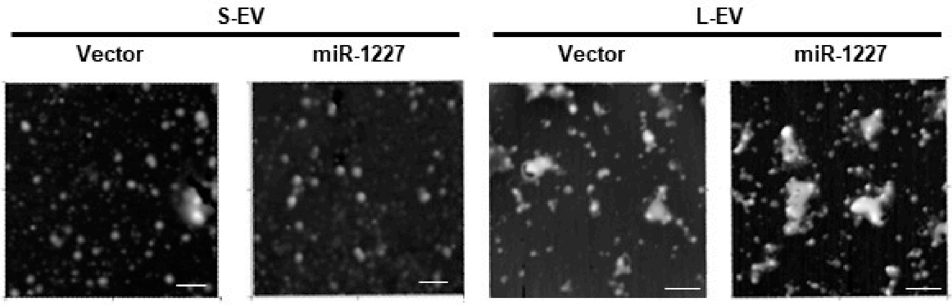

C

Input:SEC23A 3'UTR

>NM_006364.4 Homo sapiens Sec23 homolog A, coat complex II component (SEC23A), mRNA

```
TGAAGTGCTAATAATGTTAAAGACACTTAAGAAGATGAAATAATATTCAAATTTCATTTTTTCCTTTTTC
CATTTATCTGTGGAAACCAACAGATATTGCTCTATATTTTTTGTATTAGTATGGTTTGAGACAACATATG
GAAAATGTTCACATTTGTAGATTAAGCTGGAATTATAATGAGAGCAATAAGAACAAATTTATTTTGCTTA
CCACAGTGTTATAGCTGGTTCTAGAAATTTGAAGTCTTTATAACTTAATTATGTTTAATAAAAAAATAGAG
TCTGCCTCGTACTACAGATGTAACTCATTTGTATATTGCAGACAGACCCAAAGTGGCACTGAATTTTCTT
GCTCACCTTTTAAAAACTTGTTCCTTAATTTTAGCCAGAAAGCAAAAAAACAATAGTAATGATAAATGTG
AACATTTTTGCTTATTCATTGAATATTTTTCTGTAATTTTCAGCACTTATGTATACACTTTTTCTGTACT
TACTAGGTTAAGGCAGATTTATTTTTATGATTTGTTTAGGAATTATTTGATTTTATAATGGTAATTTTCA
TGATGATAATGTTTTTGGTTATTTGGAAAGATAGTTTAGAGATGAAAGGTTTTTTTGGGTAACAATCCCG
CAGCTGACAAAAAATGTGAAATTTCCACAAAATATCCAACTTATGTGACTAAACGCAGTAGTTTTTTTAA
AAGGGGAGATAGAAAATAAATGGTTTTGTTGGAGTGCATTTTAGTAAGCCTTTGCAGTAAAATGACGGTT
GTAACTACTAAACCAAATTTAGTTTTCACAGCATGGTTTTGTTGTTTTCCCCTTGTTTTTCAGAGGTAAA
TTTTGCATTATATCCTTCAGTATTTTAACACTATTTTGGCAGTTTACACATTACTTTTTGTTTTTCCTTC
CTTTTTGTGAAATGTATTAAGTTGTGGTTCTTATTGAAACAGTATTATATAATGTTTGCTTAATTATATC
ATGTGATGCTCAGTTCTATTTTGATTTATTCATTAGTATTCACTTTTACCTTTAAAGTTTACTTGTAGCA
AATATGTTTACATTGATAAAGCCAGATATGTTTTGACAATGAAATTTACATATCAAGTACTGCAAATAAA
AGGTGGTGCTATGATATATGCTTAGGAGGACAGTTTTAATGATTGTACTTGCATGAACACAATCATATGA
TGGTAAAGCAGAAACTTAAGAAAAAAATTGTTTATGTGTTATATTCAATTAGCTTAAATAAGTTGCTTTGT
TATATTTTATTTGAATTGAACTACGCTAGGCCTAAATGCCAATAAAATATACTTTTCACTGTT
```

miR-1227 binding site A

miR-1227 binding site B
