# Supplementary material for: miR-1227 Targets SEC23A to Regulate the Shedding of Large Extracellular Vesicles
Source: Cancers (Basel). 2021 Nov 22;13(22):5850. doi: 10.3390/cancers13225850 (PMC8616086; doi:10.3390/cancers13225850)
Supplement: Supplementary file 1 [file cancers-13-05850-s001.zip › Supplementary Figure S2 .pdf]

**Figure 3B**

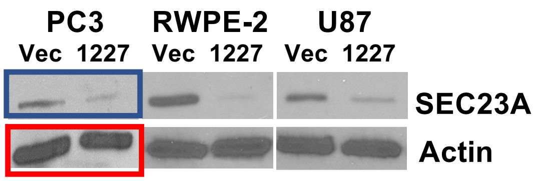


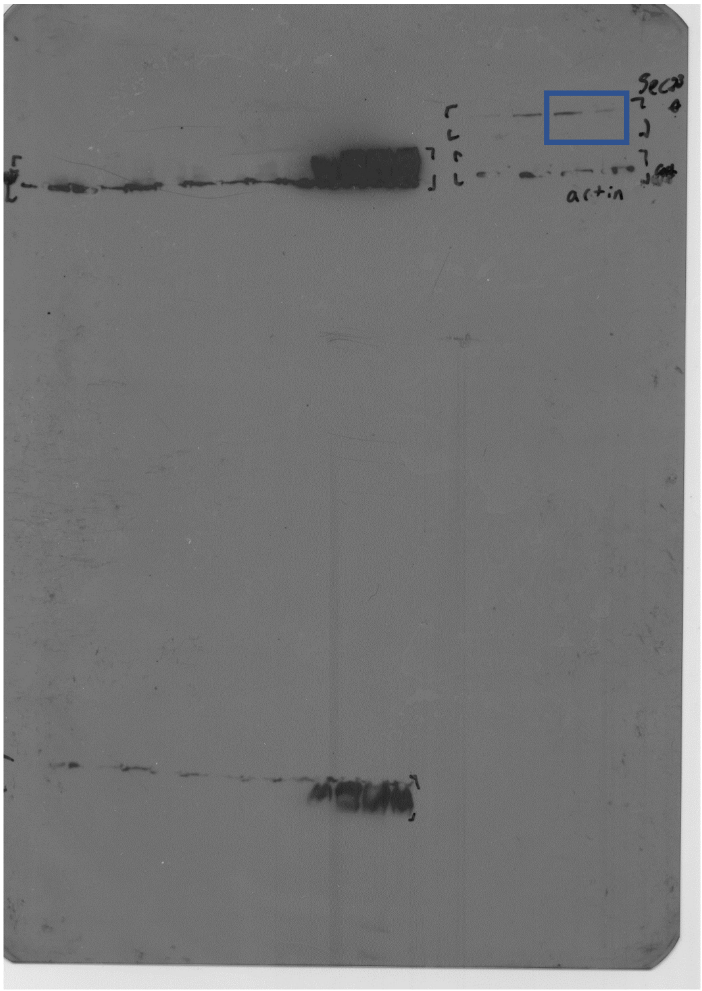


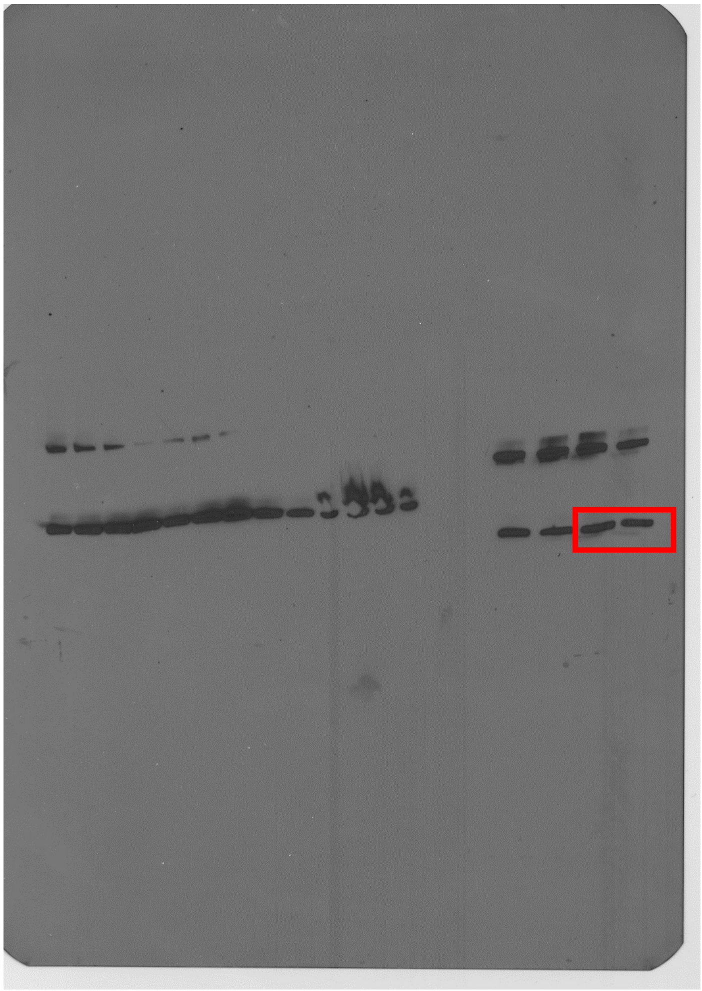

2018-08-02 actin short0001

2018-07-30 PC3 cells treated short0001

Figure 3B

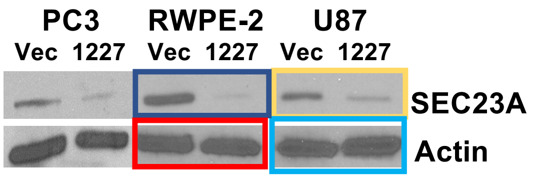


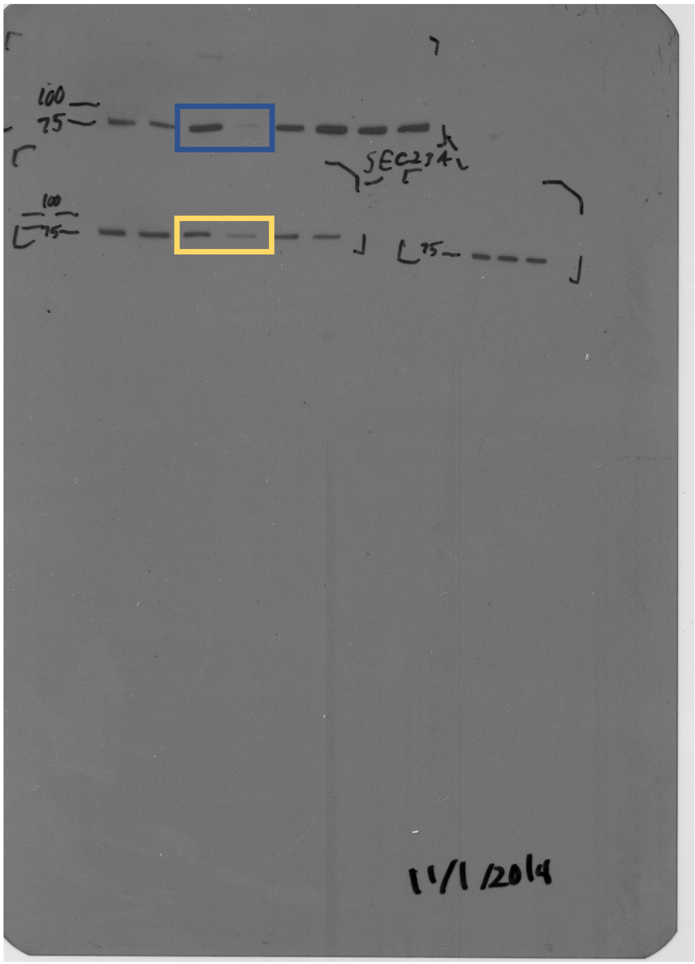

2018-11-01 RWPE-2 PC3 U87 stable cells tech1 SEC23A0001.tif

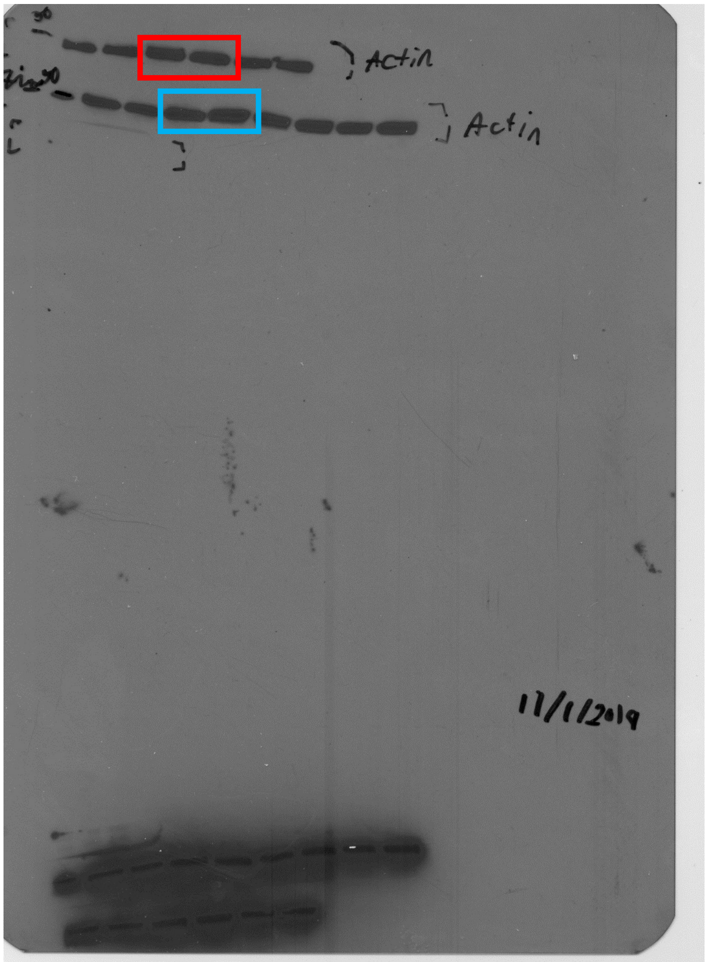

2018-11-01 RWPE-2 PC3 U87 stable cells tech1 actin0001.tif

**Figure 4C**

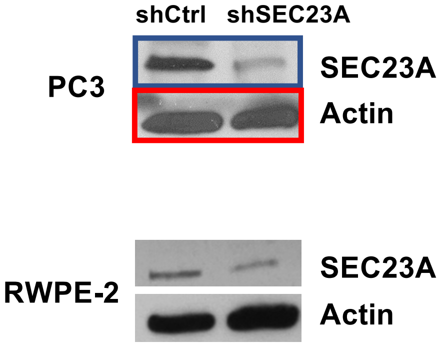


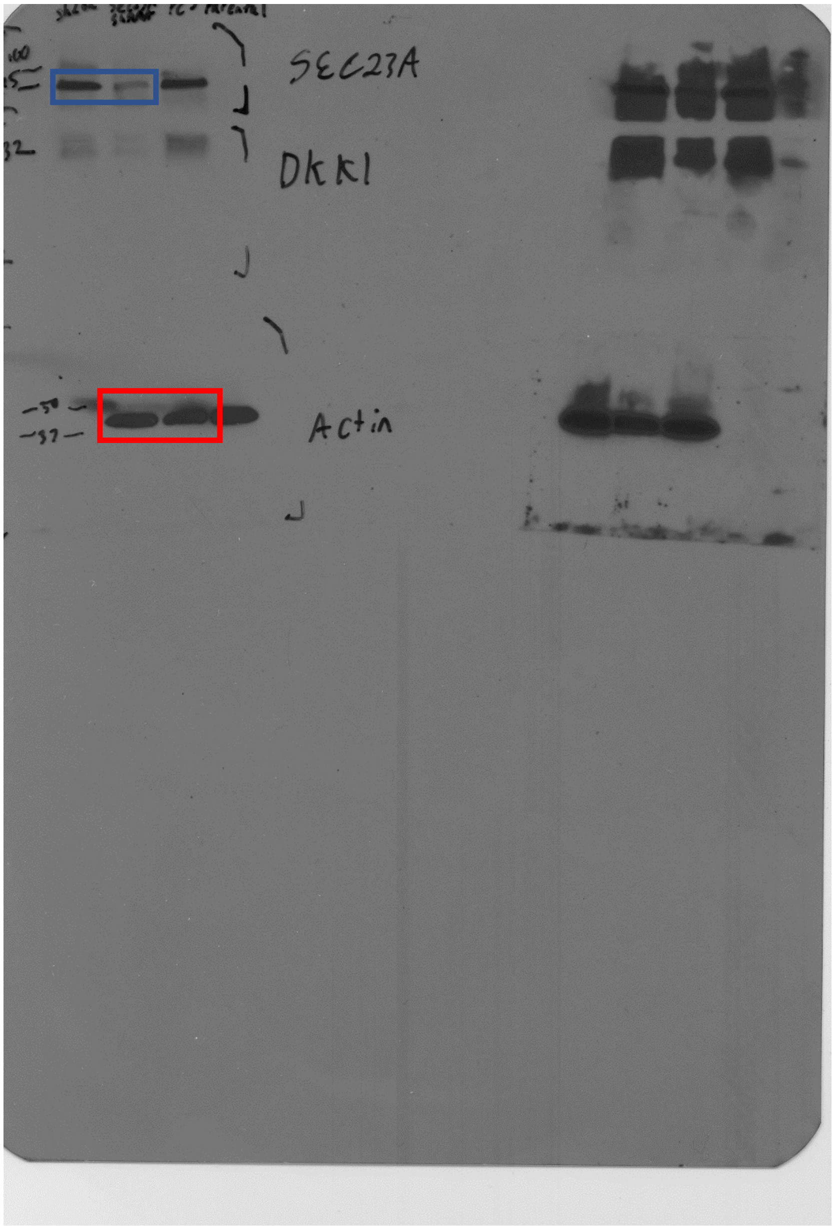


2018-11-30 PC3 SEC23A shRNA Wblot0001

## Figure 4C

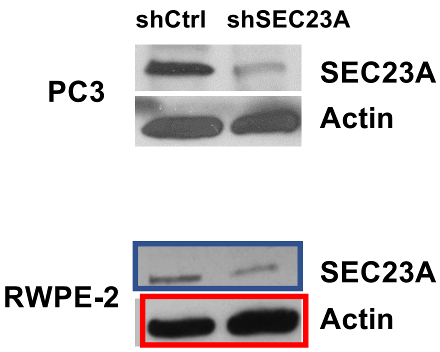


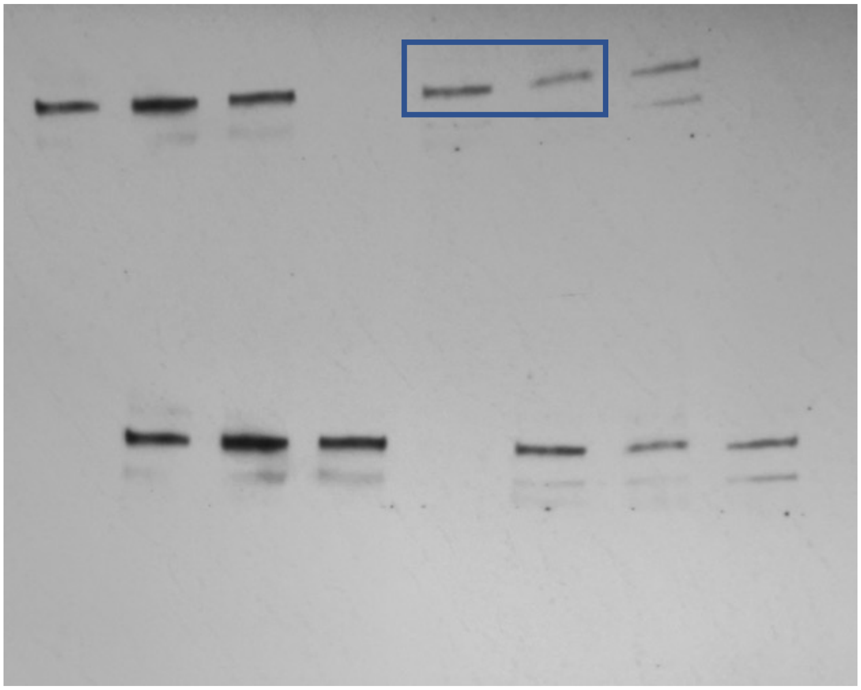

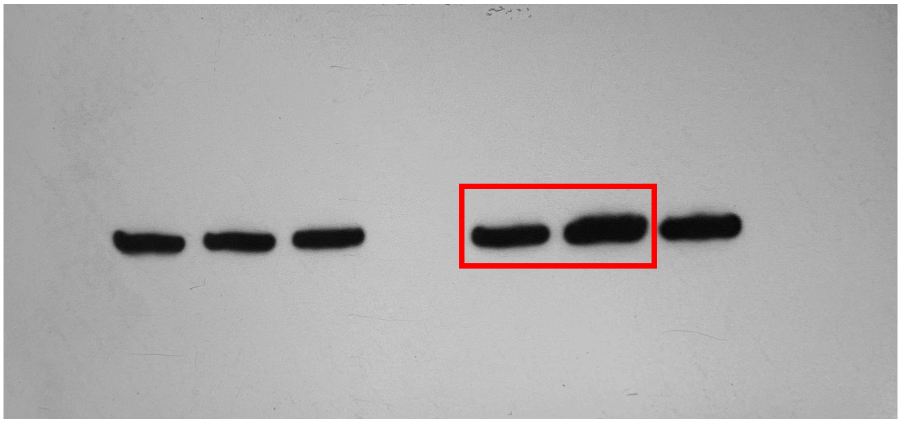

**Figure 4J**

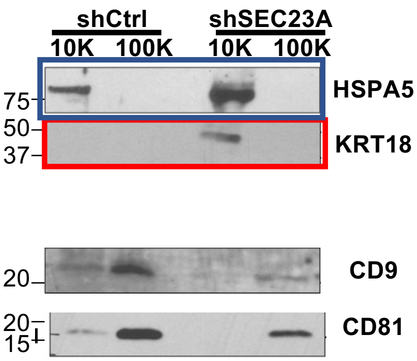


200530 Andys CD9 HSPA5 2

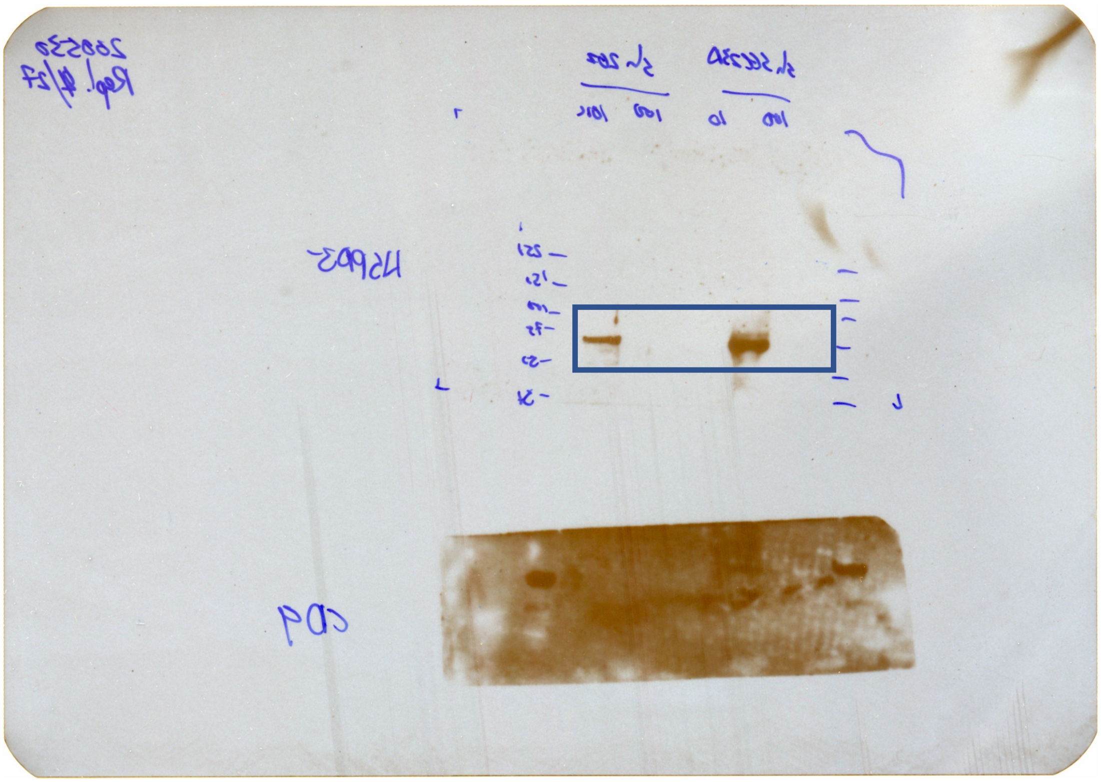

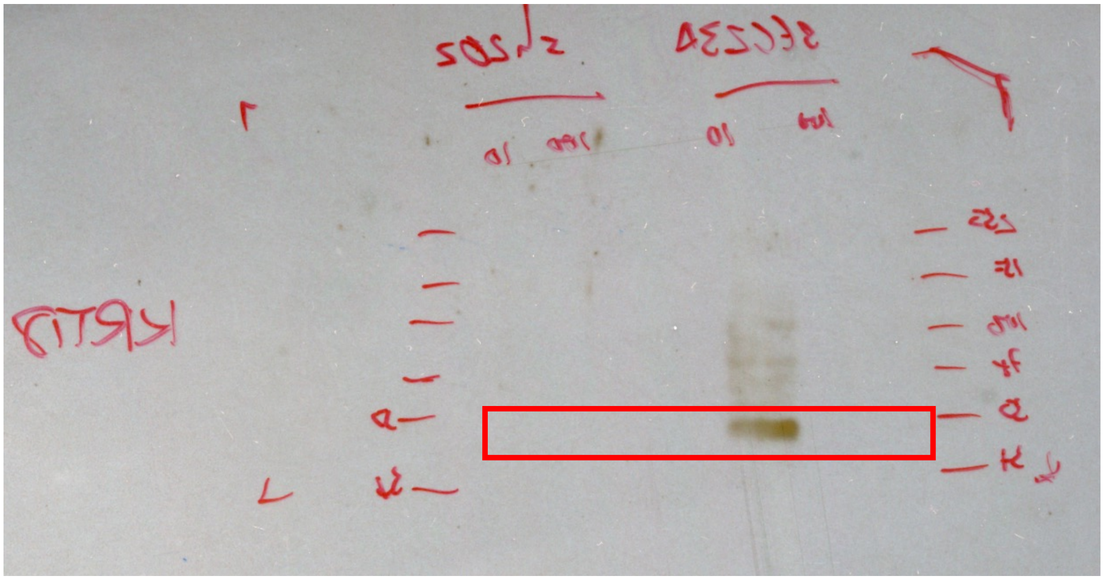

210313 Andys

## Figure 4J

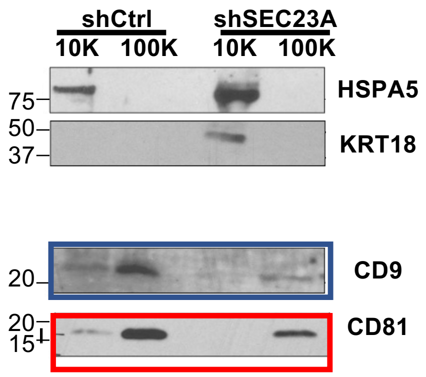


200529 CD9 HSPA5 4

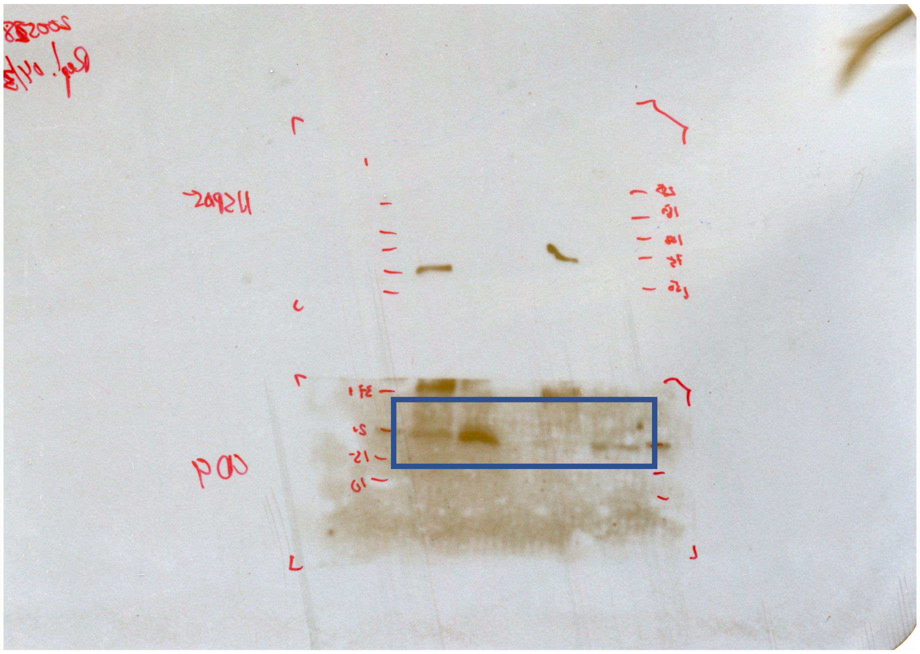

200529 CD81

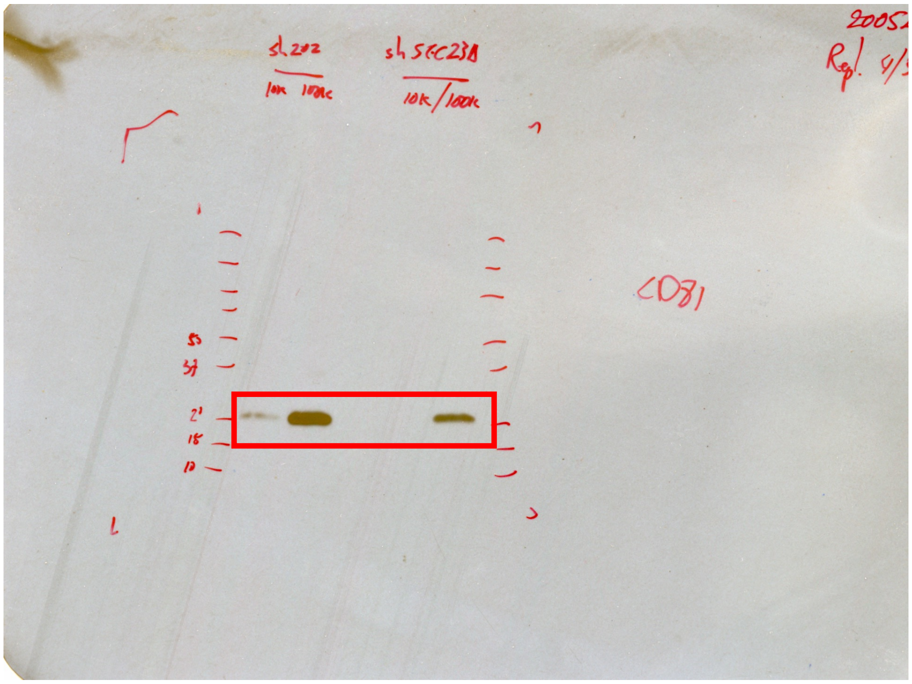

**Supplementary Figure 1A**

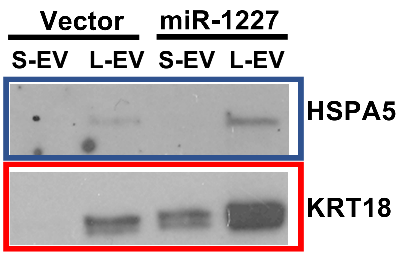


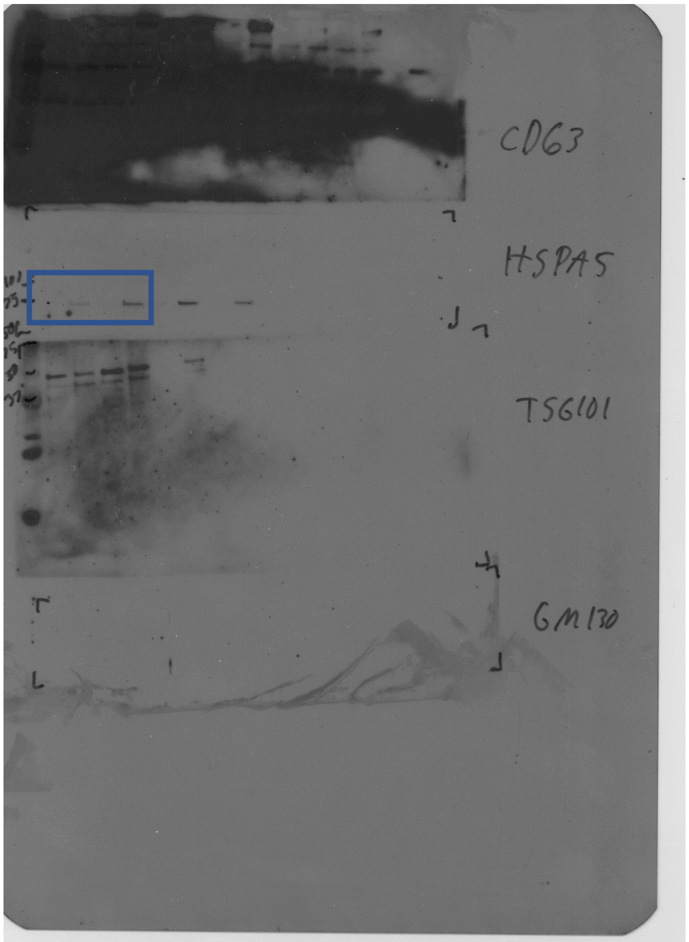


2018-05-14 PC3 and U87 EV HSPA50001

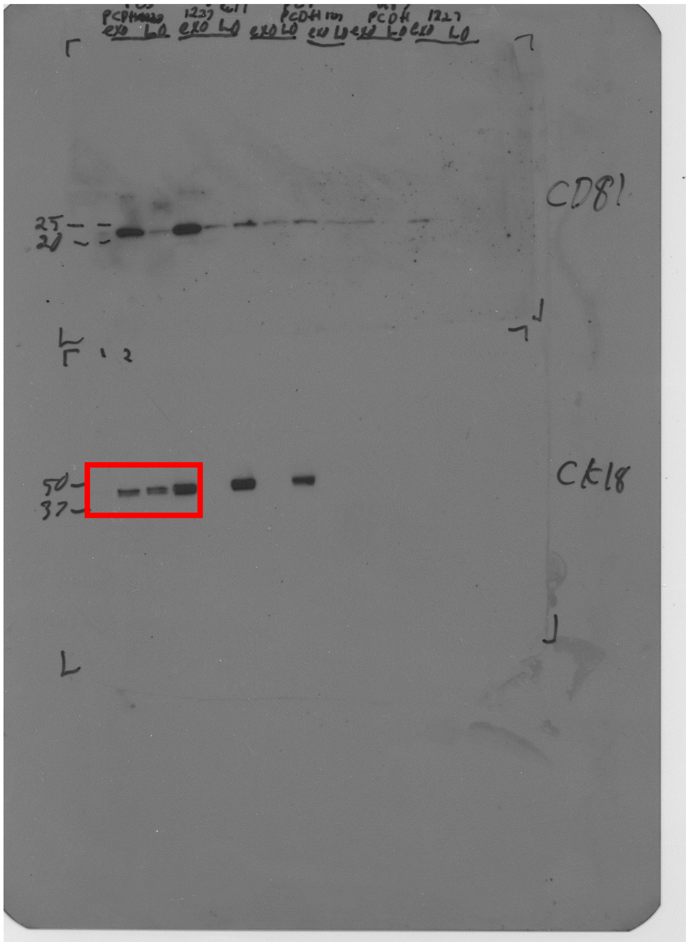


2018-05-14 PC3 and U87 EV CD81 and CK180001
